# Small Bowel Diverticulitis: An Unusual Cause of Abdominal Pain

**DOI:** 10.5334/jbsr.3540

**Published:** 2024-03-18

**Authors:** Dunkan Petersbourg

**Affiliations:** 1 Université libre de Bruxelles, Belgium

**Keywords:** small bowel, diverticula, diverticulitis, Jejunal diverticulitis

## Abstract

*Teaching point:* Small bowel diverticulitis, much less common than its colonic counterpart, is a diagnosis that must be considered in the presence of abdominal pain, especially in an elderly person.

## Case History

**Figure 1:**
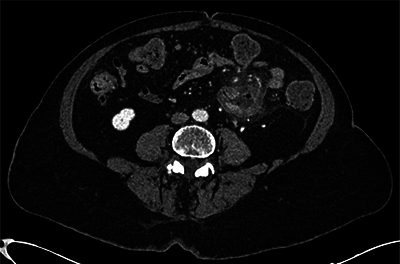
Axial contrast-enhanced CT image revealing jejunal diverticulitis without complications.

**Figure 2:**
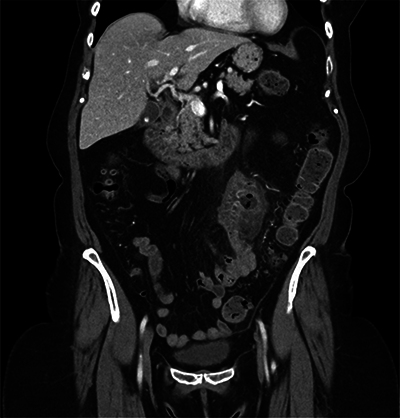
Coronal contrast-enhanced CT scan highlighting uncomplicated jejunal diverticulitis.

**Figure 3:**
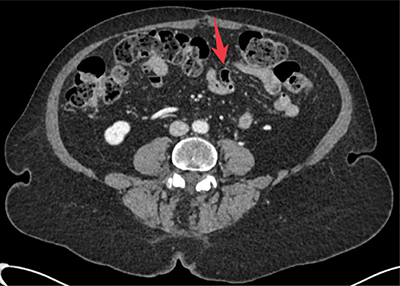
Axial CT image showing jejunal diverticulum from prior examination.

A 75-year-old female patient was urgently referred to the emergency department by her family doctor due to diffuse pains, primarily located in the abdomen. Clinical and emergency examinations revealed diffuse abdominal pain, particularly intense in the left iliac fossa, along with a biological inflammatory reaction characterized by a C-reactive protein (CRP) level of 151 mg/L. Given the patient’s multiple history of sigmoid diverticulitis, an abdominal computed tomography (CT) scan was performed to exclude a recurrence. The examination revealed uncomplicated jejunal diverticulitis (Figures 1 and 2). The jejunal diverticulum in question was retrospectively identifiable in previous examinations (Figure 3).

## Comments

Diverticula can develop throughout the gastrointestinal tract and affect, less frequently than their colonic counterparts, the duodenum, esophagus, stomach, and finally the jejunum and ileum, in descending order of frequency. It is estimated that small intestine diverticula are present in approximately 2% of the population, categorized as acquired and congenital types.

Acquired small intestine diverticula form on the mesenteric edge of the intestine, where blood vessels penetrate the intestinal wall, creating a weak area. High intraluminal pressures, resulting from a low-fiber diet or peristaltic defects related to abnormalities in the myenteric plexus, can lead to the formation of pseudodiverticula, containing only the mucosa and submucosa. These pseudodiverticula primarily occur in men over 40 years of age.

Congenital diverticula are predominantly represented by Meckel’s diverticulum, which results from incomplete regression of the omphalomesenteric duct and is located on the antimesenteric side of the ileum, approximately 80 cm from the ileocecal valve [1].

Approximately 10% of small intestine diverticula are associated with complications, with diverticulitis being the most common. Symptoms are often nonspecific, mainly manifesting as diffuse abdominal pains.

CT scanning is a key diagnostic tool, revealing diverticular formations with segmental thickening of the adjacent small intestine wall and fat infiltration. Distinguishing it from a malignant etiology can be challenging, but vascular engorgement and the presence of fluid in the mesentery are more suggestive of a diverticular origin, as well as the absence of contiguous lymphadenopathy [1].

Small-intestine diverticulitis should be considered as a potential diagnosis, given that the mortality rate can reach up to 40% due to delayed diagnosis and the often advanced age of the affected patients.
